# Morphometric and Nanomechanical Features of Platelets from Women with Early Pregnancy Loss Provide New Evidence of the Impact of Inherited Thrombophilia

**DOI:** 10.3390/ijms22157778

**Published:** 2021-07-21

**Authors:** Tonya Andreeva, Regina Komsa-Penkova, Ariana Langari, Sashka Krumova, Georgi Golemanov, Galya B. Georgieva, Stefka G. Taneva, Ina Giosheva, Nikolina Mihaylova, Andrey Tchorbanov, Svetla Todinova

**Affiliations:** 1 Institute of Biophysics and Biomedical Engineering, Bulgarian Academy of Sciences, Acad. Georgi Bonchev, Str. Bl. 21, 1113 Sofia, Bulgaria; t_andreeva@abv.bg (T.A.); arianalangari@abv.bg (A.L.); sashka.b.krumova@gmail.com (S.K.); sgtaneva@gmail.com (S.G.T.); ina_gi@abv.bg (I.G.); 2 Department of Biochemistry, Medical University, 1 St. Kliment Ohridski Str., 5800 Pleven, Bulgaria; rkomsa@gmail.com (R.K.-P.); g.golemanov@abv.bg (G.G.); galiag_77@abv.bg (G.B.G.); 3 University Obstetrics and Gynecology Hospital “Maichin Dom”, 2 Zdrave Str., 1463 Sofia, Bulgaria; 4 Stefan Angelov Institute of Microbiology, Bulgarian Academy of Sciences, Acad. Georgi Bonchev, Str. Bl. 26, 1113 Sofia, Bulgaria; mihaylova_n@microbio.bas.bg (N.M.); tchorban@microbio.bas.bg (A.T.)

**Keywords:** platelets, early pregnancy loss, atomic force microscopy, polymorphisms in thrombophilia genes, flow cytometry

## Abstract

Pregnancy is associated with hypercoagulation states and increased thrombotic risk, especially in women with thrombophilia. We combine atomic force microscopy (AFM) and flow cytometry to examine the morphology and nanomechanics of platelets derived from women with early pregnancy loss (EPL) and control pregnant (CP) and non-pregnant (CNP) women. Both control groups exhibit similar morphometric parameters (height and surface roughness) and membrane stiffness of platelets. EPL patients’ platelets, on the other hand, are more activated than the control groups, with prominent cytoskeletal rearrangement. In particular, reduced membrane roughness (22.9 ± 6 nm vs. 39.1 ± 8 nm) (*p* < 0.05) and height (692 ± 128 nm vs. 1090 ± 131 nm) (*p* < 0.05), strong alteration in the membrane Young modulus, increased production of platelets’ microparticles, and higher expression of procoagulant surface markers, as well as increased occurrence of thrombophilia (*FVL*, *FII20210A*, *PLA1/A2*, *MTHFR* *C677T* or 4G/5G *PAI-1*) polymorphisms were found. We suggest that the carriage of thrombophilic mutations triggers structural and nanomechanical abnormalities in platelets, resulting in their increased activation. The activation state of platelets can be well characterized by AFM, and the morphometric and nanomechanical characteristics might serve as a new criterion for evaluation of the cause of miscarriage and offer the prospect of an innovative approach serving for diagnostic purposes.

## 1. Introduction

Platelets are small (1–2 μm in diameter) anucleate blood cells that have a discoid shape and smooth surface in a resting state. In response to various biological stimuli and agonists, vascular damage or shear stress, they undergo dramatic changes in their morphology and size, due to the reorganization of their cytoskeleton, a process known as platelet activation [1,2]. The first event that takes place is related to a change in cells’ shape from discoid to spherical, followed by prominent cell–cell interactions and clustering through extending actin-rich philopodia; in the final phase, flattening and spreading of cells on the damaged surface is observed, which results in the sealing of the impaired vessels. More precisely, five stages of spreading and activation of platelets can be distinguished: (1) round or discoid without pseudopodia; (2) dendritic with early pseudopodia but without flattening; (3) spread-dendritic with intermediate pseudopodia (one or more flattened pseudopodia) without spreading of the cell body; (4) initial spreading with late pseudopodia; (5) fully spread without distinct pseudopodia [3,4]. Abnormalities in platelets size and shape, as well as in the activation process, are related not only to impaired haemostasis but also to various pathologies and inherited platelet disorders [5,6]. Platelets are also involved in metastasis, various inflammatory processes, innate and adaptive immune defences, and embryonic development [7]. Therefore, the investigation of platelets’ activation is essential for understanding the regulation of blood coagulation in healthy and diseased individuals. This is particularly important for pregnancy, which is defined as a hypercoagulable state.

The balance between the pro- and anticoagulation system is strongly altered during normal pregnancy, when procoagulant effects become dominant [8,9], which is a stimulus for the activation of platelets. The changed balance leads to maintenance of a stable placental function during pregnancy and prevents per partum blood loss. It was demonstrated that platelet reactivity varies according to gestational age and agonist [10,11]. In this regard, P-Selectin (CD62p), a well-known biomarker for platelet activation that is secreted by degranular platelets, is found to increase with gestation [12]. Sheu and colleagues demonstrated that platelet aggregation and ATP release via collagen and adenosine 5’-diphosphate stimulation were markedly enhanced in platelets isolated from pregnant subjects compared to those from nonpregnant controls [13].

Several of the most common complications during pregnancy, such as preeclampsia, placental abruption, intrauterine growth retardation or fetal death, are due to abnormal placental vasculature and haemostasis disorders, which lead to impaired maternal circulation. Activated protein C and free protein S sensitivity ratio (APC:SR) showed a progressive fall through pregnancy, which correlated with changes in *factor VIIIc* and *factor Vc* [14].

It is believed that pregnancy could play an important role in triggering early clinical manifestations of hereditary forms of thrombophilia [15] and, therefore, the effect of thrombophilia on pregnancy and early pregnancy loss (EPL) occurrence has been actively studied in recent decades [16,17,18,19]. Prothrombotic mutations (inherited thrombophilia) may induce placental hypercoagulation and lead to the formation of microthrombosis in utero-placental blood vessels with subsequent ischemia, vasoconstriction and endothelial dysfunction [20]. This thrombogenic mechanism has an inhibitory effect on the process of implantation and hormone production and can lead to recurrent miscarriage in the first trimester. It is believed that inherited thrombophilia is one of the major causes of placental damage by inducing arterial and/or venous thrombosis at the site of implantation or in the placental blood vessels [21]. These processes are regulated by the endothelium, the platelets and fibrinolytic plasma proteins.

Prothrombotic mutations/polymorphisms, including FV Leiden mutation, *C677T* polymorphism in the methylene tetrahydrofolate reductase (MTHFR) gene, and *G20210A* polymorphism in the prothrombin (Factor II) gene, are suggested to induce placental hypercoagulation and to lead to microthrombosis formation in uteroplacental blood vessels with subsequent ischemia, vasoconstriction and endothelial dysfunction [20]. Carriage of polymorphisms *4G/5G* in the *PAI-1* gene and *PLA1/PLA2* in *glycoprotein IIb/IIIa* gene could also contribute to pro-inflammatory and pro-thrombotic complications. However, the information about the impact of all these polymorphisms is controversial [22,23,24]. The thrombogenic events produce an inhibitory effect on the process of implantation and hormone production and can lead to recurrent miscarriage in the first trimester. It is believed that inherited thrombophilia is one of the major causes of placental injury by inducing a thrombosis at the site of implantation or in the placental blood vessels [21]. The endothelium, the platelets and fibrinolytic plasma proteins regulate these processes [25].

The thrombotic nature of the placental vascular lesions associated with the existence of thrombophilia suggests a cause-and-effect relationship between inherited and acquired thrombophilia and the listed severe obstetric complications. For example, it was found that *FVL* and *MTHFR* mutations are significantly higher in women with preeclampsia as compared to control subjects, while the prothrombin gene mutation is significantly more prevalent in women with abruptio placentae and second trimester loss [26].

Besides the platelet-related markers with diagnostic significance that are traditionally utilized in clinical practice, such as platelet count, size, shape, mean platelet volume, platelets’ distributed width, and plateletcrit [27], in recent years, different surface expressed receptors involved in cell signalling are also recognized as reliable biomarkers. For example, increased levels of CD41 (integrin αIIβ) and CD61 (integrin β3) are found for activated platelets, while CD62p (P-Selectin) is detected exclusively in activated platelets and not in resting ones [28,29].

An important aspect of platelet activation is the release of microparticles (MPs), pro-inflammatory and pro-coagulant vesicular fragments derived from the cell membranes. Microparticles could be generated after cell activation or in the apoptotic process and released into the body fluids [30]. An increased level of MPs is observed in various clinical situations, e.g., a high level of endothelial microparticles is observed in heart failure, cancer cell microparticles is observed in cancer, and platelet MPs (PMPs) is observed in thrombosis [31]. The surfaces of PMPs contain negatively charged phosphatidylserine, which promotes the aggregation of prothrombin complexes, which in turn are essential for coagulation. Phosphatidylserine expression can activate factor X and prothrombin, which participate in intrinsic and extrinsic coagulation by recombinant factor VIIa. In addition, the release of PMPs can increase the production of adhesion molecules [32,33].

PMPs have a clinical significance because they have been shown to possess thrombogenic potential and have emerged as a possible new marker for cell/platelet dysfunction and thrombosis risk [34], as well as of other disorders including atherosclerosis, acute myocardial infarction, sepsis, sickle cell disease, cancer, diabetes and pre-eclampsia [35,36,37,38].

Despite the improvements in the diagnostic and therapeutic methods, pathologies such as miscarriage, stillbirth, preeclampsia, fetal growth retardation and placental abruption remain idiopathic. Reliable methods for prognosis and prevention of such complications of the pregnancy are limited. Therefore, new approaches and markers for early diagnosis are of utmost importance for contemporary medicine and healthcare.

Flow cytometry is among the standard techniques for the detection of platelets’ pathological activation [39] since it is able to reliably distinguish resting from activated and healthy from diseased cells from a minimal quantity of blood sample. It is also widely used for PMPs counting and characterization based on surface-exposed specific platelet-related markers [40]. Atomic force microscopy (AFM), on the other hand, is not yet well elaborated for those purposes. In this work, we explore its potential since it offers both nm resolution of the surface morphology as well as local nanomechanical characterization of individual cells in resting and activated state that can be directly correlated with the cytoskeletal morphology [41]. Girasole et al. demonstrated that cells’ roughness is a morphology-related characteristic that is independent of the overall geometric shape [42]. On the other hand, this parameter depends on the platelets granulation process—at a late stage of activation, platelets’ granules release their content in blood plasma through exocytosis, followed by fusion with the open canalicular system and a respective increase in the platelet spread area by two- to three-times [43]. The enhanced release of PMPs and vesicles from cells is one of the reasons for platelets’ bubbling and consequent change in plasma membrane roughness.

AFM has already been applied to examine the mechanical characteristics and three-dimensional changes that occur in platelets’ structure and PMPs upon activation [44,45,46,47,48]. Our recent studies have demonstrated altered morphology (shape, size and roughness) and mechanical properties of the platelet membrane in patients with deep vein thrombosis compared to healthy subjects, and the role of prothrombotic polymorphism in the ITGB3 gene of glycoprotein IIb/IIIa in the extent of these changes [49]. Altered platelet and fibrin clot network morphology was found in patients with transient ischemic attacks [50], patients with hypertension [51] and subjects with type 2 diabetes mellitus [52]. A significant change in the mechanical properties of platelets isolated from acute myocardial infarction individuals, compared to the control subjects, was also observed [53] Platelets’ shape, volume, surface roughness and cytoplasmic extensions, as well as the number and morphology of the secreted PMPs, could be useful in the identification of novel markers, which, when combined with other conventional biomarkers (e.g., histological and immunohistochemical), could optimise clinical decisions [54,55].

For this purpose, we investigate the interrelations between the polymorphisms in thrombophilia genes, the morphology and mechanical properties of platelets and surface marker expression on both platelets and PMPs for the EPL patient group, as well as for healthy pregnant and non-pregnant women. We demonstrate that the structural and nanomechanical changes of platelets derived from patients with EPL correlate well with platelet hyperactivity and carriage of polymorphism in specific thrombophilia genes.

## 2. Results

### 2.1. Main Patient Characteristics and Blood Clinical Parameters Derived for the Participants under Study

In this study, we compared the clinical and biophysical parameters of platelets isolated from volunteer women with early pregnancy loss (EPL), with those of age-matched healthy non-pregnant (CNP) and pregnant (CP) controls. The EPL group was further split into two subgroups depending on the pregnancy development: (1) EPL1 consisting of women in which the spontaneous abortion occurred between 6 and 9 gestational weeks (GW) (embryonic stage) and (2) EPL2 encompassing women in which this event took place between 10 and 12 GW (placentation stage). The main characteristics (age and gestational week at the time of miscarriage) of the studied groups as well as their blood parameters (platelets count (Plt), C-reactive protein (CRP) concentration) and coagulation state markers (fibrinogen and International normalized ratio (INR)) are presented in Table 1. The main parameters for the coagulation status of patients did not differ significantly among the control and EPL groups. A statistically lower value of CRP concentration was only found for EPL1 compared to that of the CP group. The corresponding value for the EPL2 subgroup was slightly higher but without significant difference from those of the two control groups.

### 2.2. Carriage of Thrombophilia Polymorphisms

In order to accurately determine the frequency of carriage of thrombophilia polymorphisms, such as *FVL*, *FII20210A*, *677MTHFR(Т)*, *PLA1/A2* and *4G/4G PAI*-1, in healthy age-matched non-pregnant subjects, we utilized a group of 82 women and compared the data with those obtained for the EPL1 group of patients (since the CNP, CP and EPL2 groups were very scarce for statistical evaluation of mutation carriage). The data revealed increased carriage of *FII20210A* polymorphism in the EPL1 group as compared to the set of healthy controls. The prevalence of carriage of polymorphisms *677 MTHFR*(*T*), *PLA2*, and *4G/4G PAI*-1 was also higher, although with low statistical significance (Table 2).

Similarly, to the data obtained for the larger cohort of individuals, the carriage of polymorphisms in EPL1 group was higher for the occurrence of polymorphism of the allele *FII20210 A*, *PLA1/PLA2* and *4G/4G* PAI-1 than in the CNP and CP groups. In both control groups, the *FVL* polymorphism was not detected (Table S1). It should be noted that the overall number of mutation carriers in EPL women was 88%, (among them, the double carriers were 30 %) vs. 22% in the two control groups.

### 2.3. Atomic Force Microscopy on Platelets. Morphometric and Nanomechanical Characteristics of Platelets from Control and EPL Groups

For the purposes of this study, we examined the morphological characteristics of platelets derived from the three defined groups of women. More than 380 AFM images taken in contact mode were analysed to identify statistically significant differences in the morphological parameters of the cells from the studied groups. Statistical analysis was performed based on topography scans of 165 cells from the CP and CNP groups and 218 cells from the EPL group.

**Figure 1:**
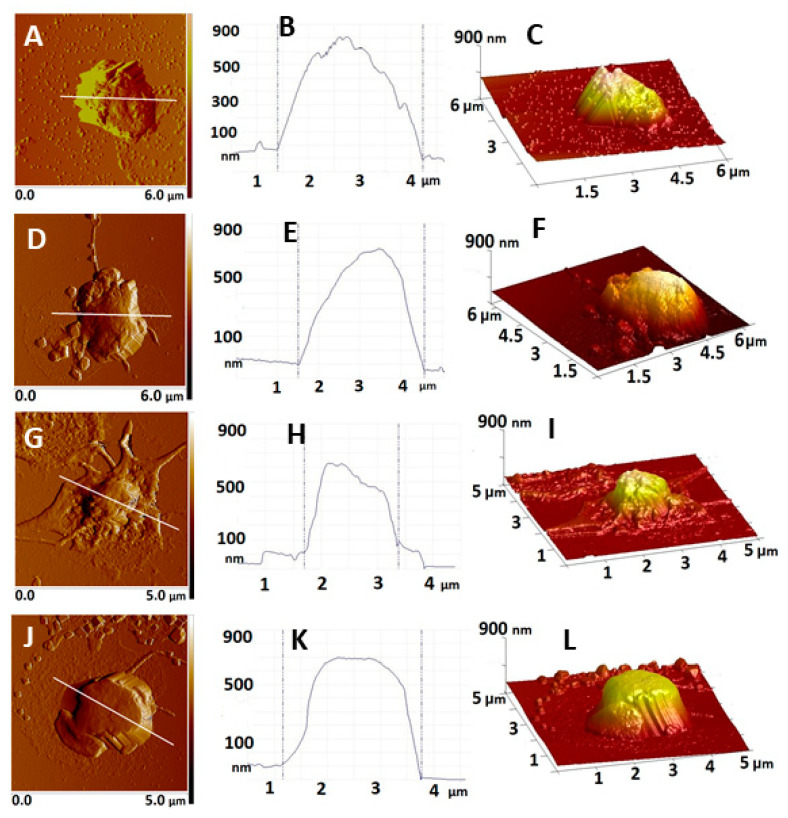
Representative 2D AFM images of platelets derived from control non-pregnant (**A**) and pregnant (**D**) women and women with early pregnancy loss EPL1 (**G**) embryonic stage of gestational development and EPL2 (**J**) placentation stage of gestational development; cross-section plot profiles (**B**,**E**,**H**,**K**) corresponding to the white lines in **A**, **D**, **G**, **J**; 3D topographical images of the images in **A**, **D**, **G** and **J**, respectively (**C**,**F**,**I**,**L**). The images were taken in tapping mode in air, at room temperature.

The AFM images presented in Figure 1 show that the cells isolated from the studied groups of women had reached different phases of activation after their spreading on the glass coverslip, induced by the contact with artificial surface. The degree of platelet activation was determined on the basis of topographic parameters such as shape, height, area and membrane roughness, as well as mechanical characteristics such as Young’s modulus.

The shape of the platelets derived from CNP women (Figure 1A) was typical for weakly activated platelets (stage 2 according to [3]), i.e., they have lost their disc-shaped profile and exhibited a spherical structure either without—or with only a few—early pseudopodia extending from the cell. Most of these cytoplasmic extensions were short and poorly formed. On most AFM images, the platelets were at a distance from each other and no interconnected platelets were observed (Figure S1A). The membrane was evenly pleated over the entire surface (Figure 1C).

The morphology of platelets isolated from CP differed slightly from that of CNP women. The cells’ shape was flatter and spread over a larger area on the glass cover slip than that of the CNP (Figure 1D). Their membrane was relatively smoother than that of the CNP (Figure 1E,F). In contrast to the CNP group, the filopodia extending from the cells were clearly visible, which is characteristic for more advanced stages of activation stage 3 according to [3]. Occasionally, the cells interacted with the adjacent ones (Figure S1B,F,G). 

Significantly different morphology of platelets isolated from women with EPL, compared to those of the two control groups, wаs observed. Extensive filopodia were the dominant feature of platelets in the EPL1 subgroup (Figure 1G). It was established that the platelets derived from the patients of the EPL1 subgroup were at a later stage of activation (corresponding to stage 5) (Figure 1G). The images showed a trend of strong adhesion of platelets to the glass cover slip in a “fried-egg style” form with broadly spread hyaloplasm and cell organelles gathered in the central part (Figure 1G,I). The filopodia formed a dense network between adjacent platelets, with most of them clustered together (Figure S1C,G,K). For the EPL2 subgroup, two populations of platelets with different shapes were distinguished. For the first type of cells (28% of the whole population), the platelets had a similar profile to that found for EPL1 (Figure S1L). The platelets’ shape in the second population was close to the cubic form (Figure 1J). The cell membrane was smooth at the central part of the platelet (Figure 1K,L). The cells were tightly clustered together with fused hyaloplasm (Figure S1D,H).

The topographic (height, cell spreading area, cell volume and membrane roughness (Rrms)) and mechanical (Young’s modulus) parameters of platelets determined from the AFM images and force curves are presented in Table 3. The statistical analysis indicated that the CNP and CP groups did not differ significantly in any of those parameters. The height of platelets derived from the EPL1 group as well as their volume was, however, significantly lower compared to that of the control and EPL2 groups (Table 3). No significant differences between the various groups were detected for the average spreading area parameter.

Cell membrane roughness (Rrms) reflects changes in the cell morphology [56] and, therefore, is an important morphometric parameter, closely related to cells’ structural integrity and the interaction of the cytoskeleton with the plasma membrane [42]. The roughness of the platelet’s membrane in the two EPL subgroups (22.9 ± 6 and 24.8 ± 8 nm, respectively) was considerably decreased compared to the control values (Table 3). 

**Figure 2:**
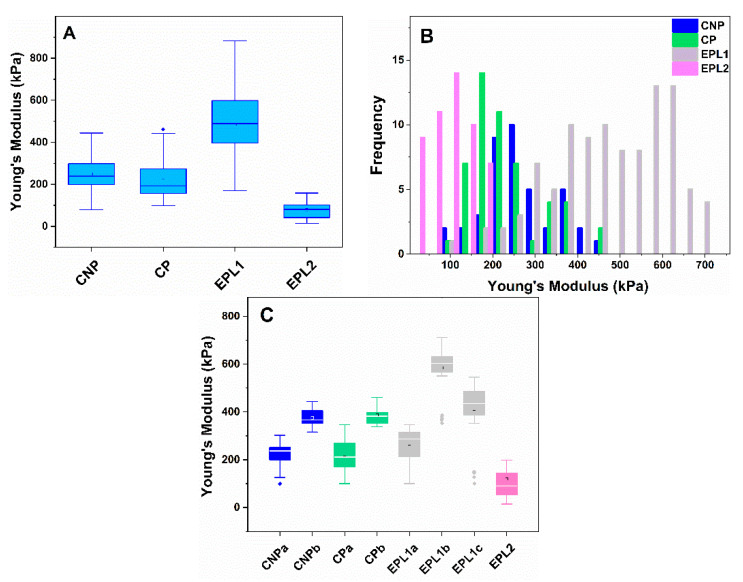
(**A**) Box plot of Young’s modulus determined for all measured cells derived from control non-pregnant (CNP) and pregnant (CP) women and women with early pregnancy loss—EPL1 (embryonic stage of gestational development) and EPL2 (placentation stage of gestational development) groups. (**B**) Histogram of Young’s modulus determined for platelets derived from the groups in panel **A**. (**C**) Box plot of the Young’s modulus determined for defined subpopulations of cells. Outliers of the interquartile range are represented by rhombi in panel **A** and **C**.

The mechanical characteristics of cell membranes were determined from the registered force–distance curves (f–d), applying the Hertz model. Figure 2 represents the box plots of Young’s modulus found of platelets obtained from women of the groups under study. It is clearly visible that the Young’s modulus of the EPL1 subgroup, determined within the interquartile range, is more than double those of the two control groups, while a significantly lower value was established for the EPL2 subgroup compared to all other groups (Figure 2A). The high dispersity of the EPL1 subgroup prompted us to study in further details the distribution of Ea values. We tested the normality of the data distribution (by Shapiro–Wilk test) and revealed that only the Ea values for the EPL2 group consisted of a single population with a normal distribution. The histogram plots of the Young’s modulus of the other groups revealed several populations of cells (Figure 2B). 

Two cell populations were distinguished in CNP and CP subjects, the dominant one with average values of 225 kPa and 221 kPa, respectively, and a second minor population with Ea centered at about 375 ± 41 kPa and 387 ± 43 kPa, respectively (Figure 2C). The mean membrane Young’s modulus of EPL1 platelets was almost twice those of the two control groups (Table 3, Figure 2A). Its corresponding histogram, however, showed a complex distribution, revealing several cell populations. The multicomponent Gaussian distribution of the data showed three sets—two largely populated ones with maxima centered at 594 ± 109 and 412 ± 114 kPa, and a smaller population with Ea of 266 ± 67 kPa, comparable to the dominant population of the CNP and CP groups (Figure 2C). It should be noted that the EPL1 group also included data determined for platelets isolated from two patients without established thrombophilic mutations. Nearly 40% of their cells exhibited Young’s modulus close to the mean values of the control groups (Ea < 300 kPa). DNA analysis revealed that the remaining patients (12) were carriers of one or two thrombophilic mutations and their Ea values were in the range of 360–710 kPa.

Even though the morphometric parameters (height and area of spreading) of EPL2 platelets did not differ significantly from those of the CNP and CP groups, the mean Young’s modulus of the cells was almost twice as low (Table 3). The distribution analysis revealed that the Ea values are centered at 97 kPa, but it should be noted that 42% of them overlapped with the lowest values obtained for the two control groups.

### 2.4. Flow Cytometry Analysis

**Figure 3:**
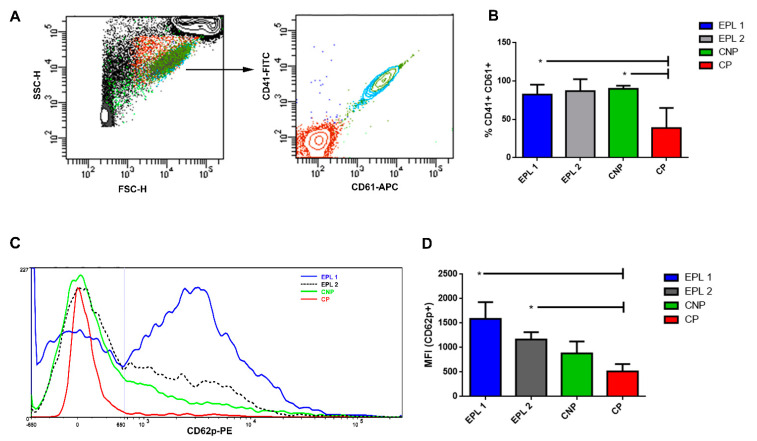
Expression of major platelet glycoprotein (CD41/CD61) and platelet activation marker CD62p. Gating strategy (**A**), percentages of platelets in the studied groups (**B**), representative fluorescence intensity (**C**) and cell count histograms (**D**) showing changes in the platelet expression of CD62p are shown; *T*-test * *p* ≤ 0.05.

In the present research, we have used flow cytometry to study the expression of platelet surface receptors both qualitatively as well as quantitatively and to document platelets’ activation in a state that is close to the physiological one. The expression of major platelet glycoprotein IIb/IIIa, including both of its subunits (CD41(αIIb, subunit α) and CD61 (ITGB3, subunit β)), was used for the gating of the different platelet populations of the studied groups (Figure 3A). The results showed that the percent of CD41 + CD61 presenting platelets’ specific marker in the EPL1, EPL2 and CNP was significantly higher as compared to the CP group (Figure 3B). A lower index in the CP group may be a reflection of mild gestational thrombocytopenia, which is relatively frequent during normal pregnancy [57].

Next, we analyzed the expression of the CD62p marker (P-selectin) on the platelets’ surface. The expression of P-selectin on the platelets’ membrane indicates the extent of the platelets’ activation state. We found a significantly elevated level of CD62p in the EPL1 and EPL2 groups as compared to the CP group (Figure 3C,D).

PMPs play a role in the normal hemostatic responses because they demonstrate prothrombinase activity. The most common marker identifying “procoagulant” platelets is fluorescently labelled annexin V, a protein that binds to the head group of anionic phospholipids such as phosphatidylserine [58,59].

**Figure 4:**
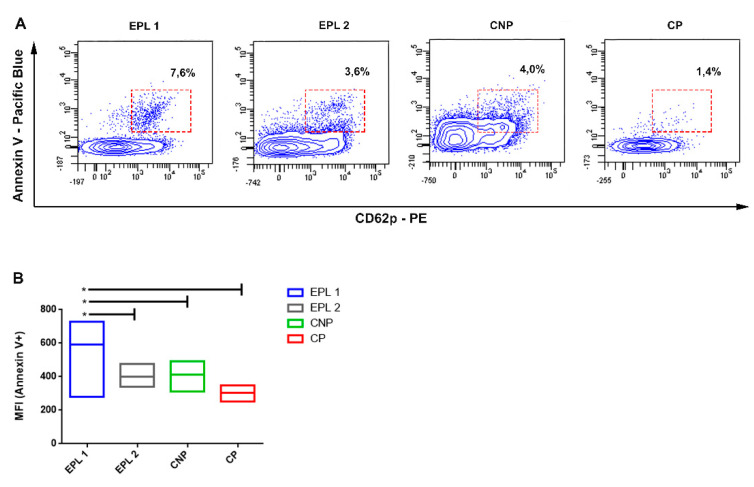
Flow cytometric analysis of platelets released microparticles in the studied groups (**A**); mean fluorescence intensity (MFI) showing changes in annexin V expression (**B**); *T*-test * *p* ≤ 0.05.

We have analyzed the levels of expression of annexin V on the surface of the CD 41 + CD62p + platelets. The results showed that the percentage of annexin V-positive PMPs in the CP group is the lowest compared to the other studied groups (Figure 4A).

**Figure 5:**
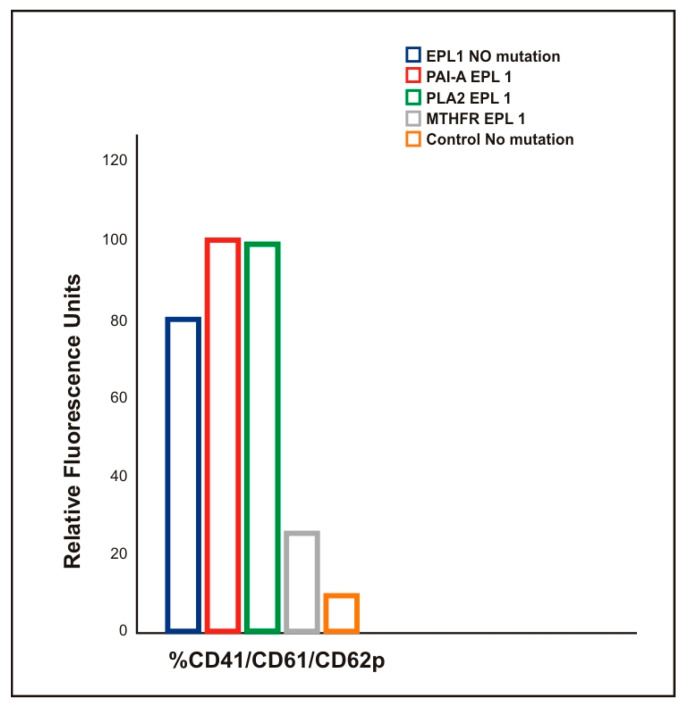
Flow cytometric analysis of platelet activation presented as MFI CD41/CD61/CD62p of the EPL1 group with respect to the carried polymorphisms compared to the control group (*T*-test *p* ≤ 0.05).

There was a statistically significant increase in EPL1 group’s fluorescence intensity as compared to all other groups that was due to higher emission from EPL1 samples and higher activation index of CD41/CD61/CD62p, particularly for the carriers of PAI-1 and PLA2 polymorphisms, as compared with EPL1 and control non-carriers of any polymorphism (Figure 5, *T*-test *p* = 0.23 and *p* = 0.10, respectively). 

The activation index CD41/CD61/CD62p of EPL1 in MTHFR carriers was lower than in the group with no mutation but higher than in the control group (Figure 5, *T* test *p* = 0.23 and *p* = 0.10 respectively).

## 3. Discussion

Miscarriage is one of the most common problems during pregnancy. In 50% of EPL cases, the etiology is not elucidated and hence they are classified as idiopathic. Therefore, the present study is directed towards the identification of novel biomarkers for EPL risk that are expected to help deal with this common problem. In particular, we provide evidence for a relation between the altered morphological and nanomechanical features of platelets and the carriage of polymorphisms in thrombophilia genes in EPL patients. We demonstrate that the AFM technique reveals new insights for EPL risk.

### 3.1. Increased EPL Platelet Activation Revealed by AFM

Recently, we have shown significant differences between the morphology and nanomechanics of platelets derived from healthy individuals and patients with deep venous thrombosis carriers and non-carriers of *PlA1/A2* polymorphism [49]. Although, in the present study, platelets were obtained from female CNP individuals within a different age range to the controls reported in our previous study, their morphological characteristics were very similar [49]. The morphometric data for CP platelets differed slightly from those of CNP, with such differences as smoother cell surfaces and the occurrence of a few filipodia suggesting slightly more activated platelets status in pregnancy (Figure 1, Table 3). However, flow cytometry data demonstrate that common activation and PMPs release markers such as CD41 + CD61 + and annexin V are even reduced in CP samples (Figure 4). Therefore, it can be concluded that normal early pregnancy is not associated with a significant increase in platelet activation and PMPs release as compared to the non-pregnant state. 

We found considerable structural and morphometric differences between platelets derived from patients with EPL and those from CNP and CP women. AFM imaging demonstrated extensive philopodia formation and cell clustering, along with reduced Rrms values (Figure S1, Table 3). Roughness is an essential parameter characterising platelets’ function that is sensitive to the cellular physiological conditions. It is known that when platelets are initially stimulated, α- and dense granules move close to the plasma membrane [60], which strongly affects the cells’ surface morphology. In resting platelets, the granules are uniformly distributed throughout the cell. The CNP platelets in this study were at the initial stage of activation since the formation of philophobia was either not observed or not well expressed. The presence of granules near the membrane should be responsible for the more pleated shape and the higher roughness value of platelets derived from the CNP and CP groups than from the EPL group. Therefore, for the studied control cells, we can assume that the cytoskeletal remodelling induced by the cells adsorption to the glass surface was still weak and the gathering of granules under the membrane contributed to its folding and the higher value of the Rrms parameter.

### 3.2. Correlation Between the Membrane Young’s Modulus and the Carriage of Thrombophilic Mutations in EPL Patients

The phase of cells’ activation depends on many factors, one of the most significant being the carrier of thrombophilic mutations. Elevated membrane Young’s modulus of platelets derived from EPL1 group patients compared to the control patients could be associated with the carriage of thrombophilic polymorphisms. It should be noted that the carriage of thrombophilic mutation in women of this group is 82%, while, in both control groups, it is only 17%. Our previous study also demonstrated that platelets of healthy individuals with the *PlA2* allele had Young’s modulus values that were almost double those of healthy individuals (noncarriers of the polymorphism), and they were also more prone to thrombotic events [49].

We found significant differences both in the cells’ shape and in Young’s modulus, determined for EPL1 and EPL2 platelets. We assume that these differences are either due to variation in the degree of platelet activation or to differences in the signal stimuli acting on them. Indeed, EPL1 platelets appeared to be more activated compared to the control and EPL2 platelets. We suggest that the cytoskeletal rearrangement in EPL2 platelets was weaker and reversible, while, in EPL1 platelets, it was stronger and irreversible. EPL2 platelets were swollen with strongly reduced membrane Young’s modulus. According to Radmacher and colleagues [44], the softer part of the cell is filled with cytosol, proteins and granules and the stiffer part corresponds to a dense network of actin and myosin filamentous. Therefore, it is very likely that the cytoskeletal structure was affected in EPL2 platelets. These dissimilarities could not be associated with the carriage of a certain type of mutations, since *FVL* and *PlA1/A2* polymorphisms were the prevalent mutations of EPL2 patients but were also found in the EPL1 group. Since the EPL2 group included patients in which spontaneous abortion took place at 10–12 GW when the placenta was already forming, it is very likely that related hormonal changes could cause these differences. Moser and colleagues established accelerated platelet consumption in the placental circulation and suggested that platelets and their cargo represent an important regulator of early human placenta development [61]. It has been observed that maternal platelets were the first maternal blood cells, which enter the intervillous space even before uteroplacental blood flow is completely established [62]. We hypothesize that in the period of initial placentation, signal pathways are triggered that counteract cell hyperactivation and contribute to the increase in platelet deformability in order to improve their ability to pass through the narrow intervillous space. We suggest that the difference in membrane Young’s modulus between the EPL1 and EPL2 groups was triggered by the changes in cytoskeletal rearrangement during a different period of gestational development. However, due to the limited number of patients studied in the EPL2 group, no definite conclusions can be drawn. Further studies with a larger number of patients are needed to clarify whether certain thrombophilic mutations, initial placental formation, or other unknown factors are the cause of the alterations observed for the EPL2 subgroup.

We found that the structural and mechanical changes of platelets derived from patients with EPL correlated with platelet hyperactivity, which is associated with the carrier of polymorphisms in the thrombophilia genes.

### 3.3. Higher Production of PMPs in EPL Patients

The results obtained by flow cytometric analysis revealed the enhanced release of PMPs from platelets derived from EPL patients who were carriers of the thrombophilia mutation compared to the control women. That was clearly demonstrated for the EPL1 subgroup, where high expression of the activation marker CD62p and the index CD41/CD61/CD62p was found for the carriers of *PAI-1* and *PLA2* polymorphisms. The activation index was higher than in the EPL1 group with no mutation and the CPN group. However, the activation index in MTHFR carriers was higher than the CPN group only (Figure 5). Flow cytometry data on the expression of major platelet glycoprotein (CD41/CD61) and the platelet activation marker CD62p demonstrate that those common activation markers were higher in CP than in CNP (Figure 3). Therefore, it can be concluded that normal early pregnancy is also associated with an increase in platelet activation as compared to the non-pregnant state.

Flow cytometry data demonstrated that PMP release markers such as CD41 +, CD62p +, and annexin V were significantly higher in the EPL1 group, but were slightly reduced in CP samples as compared to CNP samples (Figure 4). Therefore, it can be concluded that normal early pregnancy is not associated with a significant increase in PMPs release as compared to the non-pregnant state. The amount of PMPs increases in conditions associated with systemic inflammation [63]. It is known that mild inflammatory activity is involved in the development of normal pregnancy [64] and that systematic or uterine inflammation contributes to normal implantation and pregnancy [65]. Nevertheless, in cases where the inflammation becomes excessive, it can cause pregnancy complications, such as spontaneous abortion [66], and this is most probably the reason for the observed excessive formation of PMPs in the EPL1 group.

## 4. Materials and Methods

### 4.1. Sample Selection

Seventeen female volunteers (24–42 years of age) formed the EPL group, along with two age-matched control groups, i.e., six healthy non-pregnant (mean age of 36 ± 6 years, CNP group) and six healthy pregnant (mean age of 31 ± 4 years, CP group). Based on the period in which the EPL occurred, that group was further divided into: (1) the EPL1 subgroup (spontaneous abortion occurred between 6 and 9 GW) consisting of 14 women, and (2) the EPL2 subgroup (spontaneous abortion occurred between 10 and 12 GW) encompassing 3 women. The participants in this study were recruited by the Medical University, Pleven. The study was conducted according to the guidelines of the Declaration of Helsinki, and approved by the Institutional Ethics Committee of Medical University-Pleven (APPROVAL N 404-KENID 22/10/15). All subjects included provided written informed consent for the investigation.

Several criteria were used for volunteer selection and to ensure high research reliability. Women with chronic diseases (diabetes mellitus, or obstetric and endocrine disorders such as inflammatory changes in the internal genital organs) and carriers of balanced mutations that do not occur in the early period of pregnancy were excluded from the selection, along with cases of medication use that could affect the coagulation system. The CNP group included women with one or more live births, without thrombotic complications during and after pregnancy and childbirth. Females enrolled in the CP group included those with normal blood pressure, absence of proteinuria and without a history of previous miscarriage and any other pathologies.

### 4.2. Blood Collection

Blood samples were taken after obtaining informed consent from all participants involved in the study. Laboratory-based blood tests for the coagulation status (e.g., platelet count, fibrinogen level, bleeding time, clotting time and INR parameter) were performed for each patient/volunteer. 

For EPL patients, blood was drawn within 2 h before the curettage in order to exclude the possible influence of anaesthesia on the coagulation system, while, for the two control groups, this procedure was performed after morning fasting. Venous blood was collected in two 3 mL ethylene diamine-tetra acetic acid (EDTA) vacutainers (0.084 mL 15 % EDTA Becton, Dickinson and Company, USA) by using a 19-gauge needle. The blood from the first vacutainer was used for DNA analysis, and the one from the second vacutainer was used for platelet isolation, thereby minimizing the effect of thrombin traces on platelet activation.

### 4.3. DNA Analysis

The isolation of DNA from venous blood was performed by salting out proteins from non-frozen blood. The test of polymorphism in the genes of thrombophilia factors was accomplished by Multiplex Polymerase Chain Reaction (PCR) followed by hybridization with a diagnostic set Strip assay (Viennalab, Vienna, Austria) according to the manufacturer’s protocol. The DNA hybridization on a strip was based on the principle of selective amplification and detection of PCR products by immobilization on membrane carrier-specific probes. This analysis has high sensitivity and specificity, allowing simultaneous detection of a large number of mutations. The following allele polymorphisms of thrombophilia factors were examined for each participant in this study: FV Leiden, *G20210A* in Factor II; *675 4G/5G* in the PAI-1 gene; *PLA1/PLA2* polymorphism in the glycoprotein IIb/IIIa gene; *C677T* polymorphism in the MTHFR gene. Restriction analysis was performed using specific restrictases (Fermentas) for detection of the mutation as follows: MnlI for *FVL*; HindIII for *G20210*; HinfI for *MTHFR C677T*; MspI for *PlA2* in the glycoprotein IIb/IIIa gene, resulting in the creation of a restriction site [51]. Allele-specific PCR was used to detect *4G* and *5G* genotypes. Amplification was performed in two separate series, with the two constitutive primers and two inner primers corresponding to sequences *4G* and *5G* [67]. 

Along with the characterization of polymorphisms in the studied groups in this work, we also determined the polymorphisms in a larger cohort of healthy control subjects (n = 82, mean age 37.4 years).

### 4.4. Platelet Preparation and Immobilization for AFM Analysis

Blood samples were centrifuged at 150 g for 15 min. The yellowish supernatant, i.e., platelet-rich plasma, was transferred to a new tube and centrifuged at 390 g for 5 min. The supernatant containing platelet-poor plasma was removed and the pellet (platelets) was resuspended in phosphate-buffered saline (PBS, pH 7.2). After double washing in PBS buffer, an aliquot of 100 µL was deposited onto a sterilized round glass coverslip (12 mm in diameter) and incubated for 30 min. After that time, a two-step washing procedure with 1 mL of PBS buffer was applied to remove any erythrocytes that could remain in the sample suspension, as well as unadhered platelets. Next, the samples were fixed for 40 min in a 1% glutaraldehyde (pH 7.4), followed by triple washing with PBS buffer. The last step before the AFM experiment was gentle drying of the samples under nitrogen flow.

### 4.5. AFM Measurements

AFM data collection for imaging and force mapping of platelets was performed using the NanoScope V system (Bruker Inc, Mundelein, Illinois, IL, USA) coupled with an optical microscope interfaced with a CCD camera for microscopic observation of the cells over the sample surface. At first, the cells ware scanned in contact mode, in air, using a pyramidal tip with radius <10 nm, attached to a cantilever with a reflective aluminium coating of 450 µm length, 50 µm width and 2 µm thickness, with 0.2 N/m nominal spring constants (ContAl-G, BudgetSensors, Innovative Solutions Ltd., Sofia, Bulgaria). The cells were examined with a very slow scanning rate of 0.2 Hz and the images (512 × 512 pixels) were captured in height and error modes and analysed using Bruker NanoScope Analysis 1.3 software. Soft gold/chromium coated silicon nitride contact mode AFM probes (SiNi BudgetSensors, Innovative Solutions Ltd., Sofia, Bulgaria) were utilized for the force–volume mapping of the cells. The tip (radius <15 nm, macroscopic half cone angle of 35°) had a pyramidal shape and was mounted on a gold-coated cantilever with a nominal force constant of 0.06 N/m. The system was calibrated prior to each experiment by measuring the deflection sensitivity (about 52 nm/V) and force constant of the cantilever by thermal tuning using a clean glass surface. The relative force set point was set to 2 nN and 16 × 16 grid of force curves was taken at a lateral scan rate of 1 Hz.

Topographic images in contact mode were obtained to compare the morphology of the cells. Force–distance curves, recorded at the central area of each platelet, were used to calculate the Young’s modulus. Calibration of the cantilever’s deflection sensitivity and spring constant allowed the quantitative analysis of these force–distance curves.

Platelets, especially activated ones, have inhomogeneous structures and display altered viscoelastic behaviour at the different cell sections. Hence, Young’s modulus and respective elasticity varied significantly at the different parts of the cell. To yield reasonable results, the f–d curves (16 curves on an area of 0.7 × 0.7 µm) were recorded in the central part of each platelet. Young’s modulus (Ea) was determined based on the fit of the force–distance curves (f–d) to the Hertz model using AFMech Suit software [68]. In order to avoid the influence of the substrate, the indentation depth was set to 50 nm (which is less than 10% of the height of the cells, where the Hertz model is valid).

The roughness evaluation was performed using Bruker Nano-Scope Analysis 1.3 software, in a fixed area (0.7 µm by 0.7 µm) in the central part of the platelets, as its value strongly depends on the scanned area. To avoid the effect of cell distortion, we applied pre-alignment of the first row for the selected area. The R_rms_ value was defined as the mean square root of the heights distribution as follows:

$$R_{\mathrm{rms}}\;=\;{\sqrt{\sum_{i=1}^{N}\frac{{\left(z_{i}-z_{m}\right)}^{2}}{\left(N-1\right)}}}_{}$$

where *N* is the total number of data points, *z_i_* is the height of *i*-point and *z_m_* is the average height.

### 4.6. Blood Sample Preparation for Flow Cytometry

Blood samples were collected into sodium citrate anticoagulant at a 0.105 M final concentration (BD Vacutainer). The whole blood was diluted 1:10 in modified HT buffer (10 mM HEPES, 137 mM NaCl, 2.8 mM KCl, 1 mM MgCl_2_, 12 mM NaHCO_3_, 0.4 mM Na_2_HPO_4_, 0.35% (*w*/*v*) BSA, 5.5 mM glucose, pH 7.4) in the presence of 50 µg/mL GPRP (Gly Pro Arg Pro, Sigma-Aldrich). The blood sample was additionally diluted 1:1 in HT buffer supplemented with 6 mM CaCl_2_. After that 100 µL of platelet suspension was added to the bottom of FACS tube. The appropriate antibody cocktail was added to each tube. The tubes were shaken gently and incubated in the dark at room temperature for 20–30 min. The platelets were fixed with BD CellFIX™ (BD Biosciences™). Fixed samples were measured by flow cytometry (LSR II, BD Biosciences) and analyzed with BD FACS DIVA software (BD Biosciences).

For analysis of the expression of platelet surface markers, we used the following fluorochrome conjugated monoclonal antibodies: FITC conjugated anti-human CD41 Antibody (clone MEM-06); APC conjugated anti-human CD61 Antibody (clone VIPL2); PE conjugated anti-human CD62p Antibody (clone AK4). All of these antibodies were purchased from EXBIO, Praha. For analysis of the PMPs, we used Pacific Blue conjugated anti-Annexin V antibody (clone VIPL2) (EXBIO, Praha).

Fluorescent polystyrene microspheres of known sizes (0.02, 0.1, 0.2, 0.5, 1 and 2 µm) (Flow Cytometry Sub-Micron Size Reference Kit, Invitrogen™) were used as a reference to determine the platelets’ size based on FSC-A and SSC-A parameters and to set the threshold in the flow cytometer.

### 4.7. Statistical Analysis

Statistical analysis was performed to compare the morphometric and nanomechanical parameters of platelets obtained from the groups under study, based on the examination of 428 cells from 280 AFM images. All data are presented as means ± standard deviation. The type of distribution of Young’s modulus was determined using a data normality test (Shapiro–Wilk test). A non-parametric Wilcoxon test was used to determine statistically significant differences between the data obtained for EPL groups and the control groups of pregnant and non-pregnant women. Flow cytometry results are presented as mean ± standard deviation (SD). The statistical significance of differences between two groups was determined by the paired two-tailed Student’s *t*-test. The statistical analyses were carried out using GraphPad Prism (San Diego, CA, USA). 

SPSS 23 was used for the statistical analysis of the polymorphism carriage results to calculate Chi-squared, OR and Exact Fisher test values.

In all statistical tests, *p* < 0.05 was considered statistically significant.

## 5. Conclusions

In this work, we reveal ultrastructural and genetic changes in platelets isolated from EPL patients that suffered a miscarriage in gestational weeks 6–12, in comparison to control non-pregnant and pregnant women. Those are associated with increased cell activation and expressed in: (i) altered cell shape and elasticity of the platelet membrane—result of the altered reorganization of the cytoskeleton of the cell; (ii) increased production of PMPs; (iii) increased abundance of procoagulant surface markers and (iv) increased occurrence of prothrombotic polymorphisms. Based on our observations we suggest that the carriage of thrombophilic mutations triggers structural and nanomechanical abnormalities in platelets that result in their increased activation. Therefore, it is highly probable that thrombotic events are involved in the EPL of the studied patients.

Our study showed that the activation state of platelets can well be characterized by AFM and that the morphometric and nanomechanical characteristics might serve as a new criterion for evaluation of the cause of miscarriage and offer the prospect of an innovative approach serving for diagnostic purposes.

## Figures and Tables

**Table 1:** Main characteristics and clinical parameters—platelets count, C-reactive protein (CRP) and fibrinogen concentration, and International normalized ratio (INR)—determined for the groups under study.

Groups	Age	Gestational Weeks	Platelet Count × 10^9^/L	CRP (mg/L)	Fibrinogen (g/L)	INR
CNP	36 ± 6	-	318 ± 76	4.5 ± 1.1	3.2 ± 0.60	1.00 ± 0.04
CP	31 ± 4	7–12	296 ± 50	5.8 ± 0.9	3.5 ± 0.80	0.99 ± 0.05
EPL1	34 ± 8	6–9	311 ± 39	3.3 ± 0.6 **	3.9 ± 0.86	0.95 ± 0.05
EPL2	35 ± 4	10–12	323 ± 98	6.9 ± 1.3	3.0 ± 0.82	0.91 ± 0.04

** indicates statistical difference (*p* < 0.05) from the control pregnant women.

**Table 2:** Carriage of thrombophilia polymorphisms *FVL, FII20210A, 677 MTHFR, PLA1/A2,* or *4G/4GPAI-1* calculated in percentage for 82 healthy non-pregnant controls (CNP) and the EPL1 group. Chi-Squared, Fisher’s Exact Test, Odds Ratio, 95% CI, and Fisher’s Exact Test values are shown.

Genetic Factor	Carriage in CNP %	Carriage in EPL1 %	Pearson Chi-Squared	Fisher’s Exact Test	Mantel–Haenszel Odds Ratio	95% CI
*FVL*	7.8	14.8	7.381	0.018	4.954	1.426–17.210
*FII20210A*	2.6	9.8	1.906	0.063	5.022	0.775–32.537
*677 MTHFR* *(T)*	10.6	17.6	0.998	0.430	2.006	0.498–8.083
*PLA1/A2*	21.2	31.7	0.898	0.404	1.857	0.428–8.055
*4G/4G PAI-1*	20.5	26.0	2.044	0.153	1.415	0.391–4.422

**Table 3:** Morphological (height, spreading area and roughness) and nanomechanical (Young’s modulus, Ea) characteristics of platelets obtained from non-pregnant women (CNP), women with normal pregnancy (CP) and women with early pregnancy loss (EPL), presented as mean value and standard deviation.

Platelets	Height (nm)	Area (µm^2^)	Volume (µm^3^)	Rrms (nm)	Ea (kPa)
CNP	1090 ± 131	4.25 ± 1.4	0.71 ± 0.12	39.1 ± 8	241 ± 103
CP	955 ± 88	4.85 ± 1.3	0.66 ± 0.10	28.9 ± 6	174 ± 77
EPL1—embryonic stage	692 ± 128 *	3.89 ± 1.3	0.41 ± 0.08 *	22.9 ± 6 *	482 ± 131 *
EPL2—placentation stage	873 ± 153	6.02 ± 2.2	0.78 ± 0.13	24.8 ± 8 *	97 ± 48 *

* indicates statistical difference (*p* < 0.05) from the control non-pregnant women.

## Data Availability

The data are contained within the article or the Supplementary Materials.

## Supplementary Materials

The following are available online at https://www.mdpi.com/article/10.3390/ijms22157778/s1.
