# Supplementary material for: Morphometric and Nanomechanical Features of Platelets from Women with Early Pregnancy Loss Provide New Evidence of the Impact of Inherited Thrombophilia
Source: Int J Mol Sci. 2021 Jul 21;22(15):7778. doi: 10.3390/ijms22157778 (PMC8346153; doi:10.3390/ijms22157778)
Supplement: Supplementary file 1 [file ijms-22-07778-s001.zip › ijms-1262782-supplementary.pdf]

Morphometric and Nanomechanical Features of Platelets from Women with Early Pregnancy Loss Provide New Evidence of the Impact of Inherited Thrombophilia

Tonya Andreeva [1], Regina Komsa-Penkova [2], Ariana Langari [1], Sashka Krumova [1], Georgi Golemanov [2],
Galya B. Georgieva [2], Stefka G. Taneva [1], Ina Giosheva [1,3], Nikolina Mihaylova [4], Andrey Tchorbanov [4]
and Svetla Todinova [1,*]

[1] Institute of Biophysics and Biomedical Engineering, Bulgarian Academy of Sciences, Acad. Georgi Bonchev, Str. Bl. 21, 1113 Sofia, Bulgaria; t_andreeva@abv.bg (T.A.); arianalangari@abv.bg (A.L.); sashka.b.krumova@gmail.com (S.K.); sgtaneva@gmail.com (S.G.T.); ina_gi@abv.bg (I.G.)

[2] Department of Biochemistry, Medical University, 1 St. Kliment Ohridski Str., 5800 Pleven, Bulgaria; rkomsa@gmail.com (R.K.-P.); g.golemanov@abv.bg (G.G.); galiag_77@abv.bg (G.B.G.)

[3] University Obstetrics and Gynecology Hospital "Maichin Dom", 2 Zdrave Str., 1463 Sofia, Bulgaria

[4] Stefan Angelov Institute of Microbiology, Bulgarian Academy of Sciences, Acad. Georgi Bonchev, Str. Bl. 26, 1113 Sofia, Bulgaria; mihaylova_n@microbio.bas.bg (N.M.); tchorban@microbio.bas.bg (A.T.)

* Correspondence: todinova@abv.bg

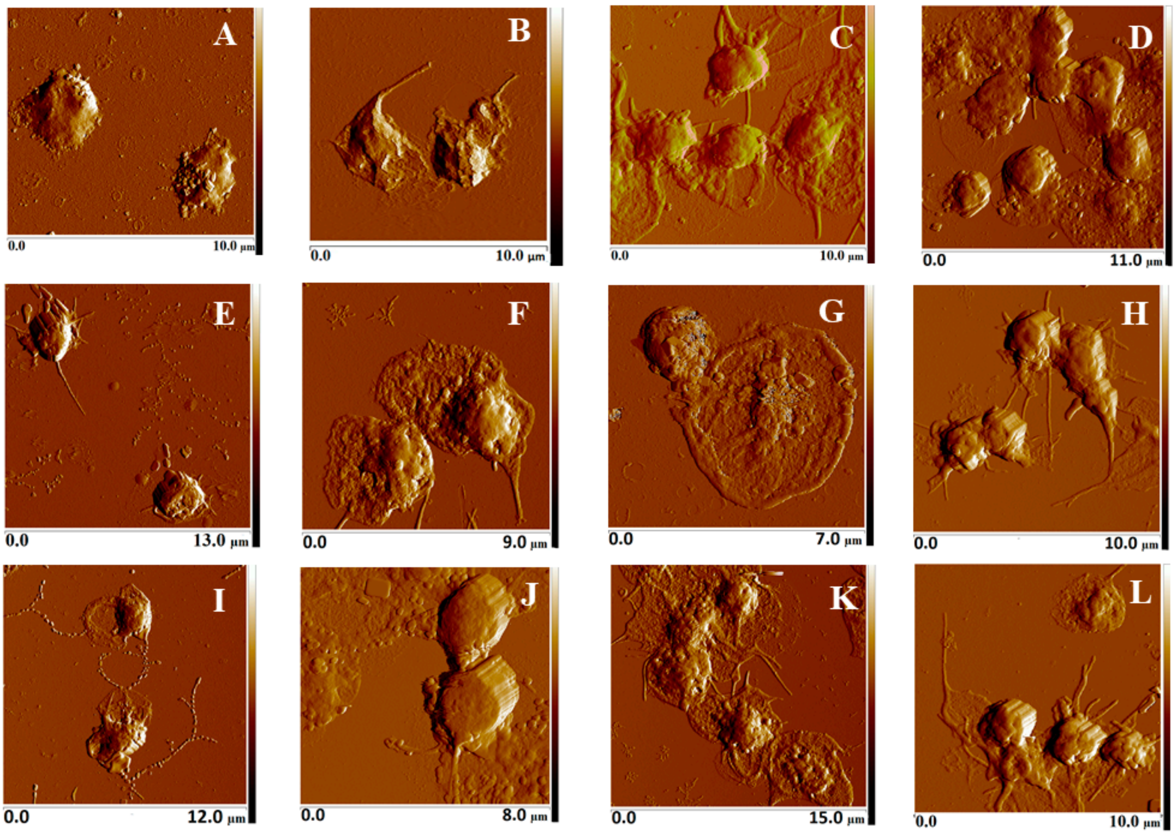

Figure S1. Representative AFM images of groups of platelets isolated from women without established pregnancy (CNP - A, E, I); control pregnant women (CP - B, F, J); women with early pregnancy loss subset EPL1 (embryonic stage of gestational development - C, G, K) and EPL2 (placental stage of gestational development - D, H, L). The images were taken in tapping mode, in the air, at room temperature.

Table S1. Polymorphism in the genes of thrombophilia factors (FVL, FII20210A, MTHFR, PLA1/A2, or 4G/4GPAI-1) determined for CNP, CP, EPL1 and EPL2 groups. Number of cases (n) of carriage of polymorphisms in the thrombophilia of genetic factors determined for each of the respective groups.

Groups	Mutations				
	FVL (n)	FII20210A (n)	MTHFR (n)	PLA1/A2 (n)	4G/4G PAI-1 (n)
CNP	-	1	-	-	1
CP			1	1	
EPL1	3	2	3	3	4
EPL2	2			1	
